# Tonic Contraction of the Lower Extremities, or Tetany

**Published:** 1886-03

**Authors:** W. H. Webb

**Affiliations:** Kingsbridge, Devon


### TONIC CONTRACTION OF THE LOWER EXTREMITIES, OR TETANY. By W. H. Webb, M.R.C.S., L.S.A., Kingsbridge, Devon.

J. K., æt. 7, a robust and healthy-looking girl, who had never been the subject of any serious illness, walked two miles to school during heavy rain on December 19th, and sat all day in wet boots. The next morning she complained of being unable to get out of bed, owing to her legs being "stiff." She remained in bed that day, and as the "stiffness" increased on the following morning, the 21st, medical assistance was sought.

I found the child in bed, lying on her left side, with the thighs flexed on the abdomen, the ankles extended, and the legs flexed on the buttocks. She was suffering no pain, and there was no tenderness. The flexor muscles of the thighs and calves were rigid, and resisted all judicious force to extend them. This condition was symmetrical on both sides, and was persistent during sleep.

The temperature in the axillæ was normal, pulse 70; no impairment of sensation, pupils normal, no sickness, clean tongue; bowels had been confined for two days.

The child was perfectly unable to stand, owing to the flexed condition of her extremities; all efforts on her part to extend them were futile. Her family history was good, and, beyond her having had measles and suffering occasionally from thread-worms, she had enjoyed perfect health. I learnt that latterly she had been suffering more than usually from these worms.

I prescribed a warm stimulating liniment, to be well rubbed into the affected muscles night and morning, and ordered her to be kept warm in bed. I gave her three powders of five grains of compound scammony with calomel, one to be taken every other night, and put her on slop diet.

Early the next morning she had a free discharge from her bowels, the evacuation teeming with multitudes of thread-worms. This was followed by a similar evacuation in the middle of the day. She was seen the next day, the 22nd, when her condition was unaltered.

On the 23rd, I found the bowels had acted similarly to the day before; the limbs were not so rigidly flexed, and I could extend them to a slight degree. From this time she gradually improved, and by the 28th had perfectly recovered. On the 25th she had prescribed liq. ferri perchlor. mit. m. v. with inf. quassiæ, three times a day.

I take it that the walk in the rain was an insignificant factor in exciting the attack, as there were no ordinary symptoms of catarrh; but that the *worms* were the real cause of the condition of this exaggerated state of reflex excitability through the nervous supply of the rectum and the muscles affected.

I have thought this case perhaps worthy of record, for the following reasons:—*Firstly*. That tonic contraction of the extremities is not a condition frequently met with. *Secondly*. That, as far as I can gather from those authors I have been able to consult, the contraction is mentioned as occurring in the upper extremities as well as in the lower, and sometimes involving the muscles of the neck and face. *Thirdly*. That the sufferers from these attacks are generally rickety, excitable, badly nourished children, and the subjects of convulsions, or laryngismus stridulus, whereas this child was the type of health.

